# Tumor-Associated Macrophages: Combination of Therapies, the Approach to Improve Cancer Treatment

**DOI:** 10.3390/ijms22137239

**Published:** 2021-07-05

**Authors:** Pedram Moeini, Paulina Niedźwiedzka-Rystwej

**Affiliations:** 1 Plant Virology Research Center, Shiraz University, Shiraz 71441-65186, Iran; pedram.moeini@yahoo.com; 2 Institute of Biology, University of Szczecin, 71-412 Szczecin, Poland

**Keywords:** cancer, macrophage, therapeutic target, tumor, tumor-associated macrophage (TAM)

## Abstract

Macrophages are one of the most important cells of the innate immune system and are known for their ability to engulf and digest foreign substances, including cellular debris and tumor cells. They can convert into tumor-associated macrophages (TAMs) when mature macrophages are recruited into the tumor microenvironment. Their role in cancer progression, metastasis, and therapy failure is of special note. The aim of this review is to understand how the presence of TAMs are both advantageous and disadvantageous in the immune system.

## 1. Introduction

The innate immune system recruits specialized mononuclear macrophage cells, with phagocytosis ability, comprising tissue-resident macrophages and circulating monocytes, located at inflammation and tissue damage sites such as tumors [1,2,3,4,5]. Tumor-associated macrophages (TAMs), the large-size macrophage cells, which infiltrate tumor tissues or tumor-like microenvironments [6], are the indispensable part of the macrophage group which serves the immune system. In fact, the tumor-condensed microenvironment, which usually includes endothelial and inflammatory cells, is not devoid of various macrophage types [7], TAMs in particular. Macrophages are reportedly the most abundant immune cells in the tumor microenvironment (TME) [8], which have been extensively investigated throughout recent decades. Their major role in cancer progression, metastasis, and therapy failure has attracted the attention of many researchers.

Decades of extensive research confirm that tumor-infiltrating macrophages critically contribute to regulating the process of tumor growth, cancer progression, and cell response to anti-cancer drugs [9,10]. The impact of macrophages on cell apoptosis, new vessel formation, and tissue inflammation is already well-established [11]. The influence of TAM functions on therapy outcomes in breast, lung, pancreatic, stomach, cervical, and many other cancers has been broadly reported [12,13,14,15,16,17,18]. Interestingly, TAMs contribute to cancer proliferation and metastasis, which has already been reported for malignant lymphoma, esophagus, breast, and liver cancers [10,18,19,20,21,22]. It has been demonstrated that TAMs create a vicious positive feedback loop of the colony-stimulating factor-1 (CSF-1)/epidermal growth factor (EGF) with tumors. The tumor cells produce CSF-1 and C-C motif chemokine 2 (CCL2) and activate TAMs. In response, TAMs secrete EGF which results in tumor cell stimulation. Breast cancer exhibits this dire feedback loop in its interaction with TAMs [23,24]. Clinical documents indicate that the high numbers of M2-type macrophages result in unsatisfying treatment outcomes and insufficient survival [14,25,26,27]. In addition, the IL-4, reportedly, destroys the effectiveness of prostate cancer radiotherapy [28]. Moreover, breast cancer tolerance to chemotherapy drugs results from the IL-6 secretion of TAMs [29]. However, the positive role of TAMs in the prognosis of pancreatic, stomach, and cervical cancers has also been reported [18,25,30,31,32,33].

Overall, in some cancers, macrophages improve the survival rate in patients and help the treatment. However, in many others, they contribute to the severe progression of cancer, especially in the deficiency of CD8+ cells [34,35,36,37]. Hence, scientists claim that they exhibit a dual role in cancer development [38,39]. Thus, despite the responsibilities of TAMs in the immune system, many researchers have focused on the major positive and negative roles they play in cancer progression because of the significance of this. Preliminary data obtained from experiments have revealed that TAM depletion nullifies their maladaptation and results in the unfavorable effects of polarization, and, thus, their critical contribution to cancer progression. Hence, recent investigations consider TAMs as an interesting therapeutic target in novel strategies for cancer treatment [40,41].

The present review outlines the advantages and disadvantages of the presence of TAMs and their impact on cancer promotion or inhibition. Meanwhile, it also illustrates TAM application in novel therapeutic strategy designs for cancer treatment, the implementation drawbacks, and future perspectives as well.

## 2. M1- and M2-Like Macrophages

There are two polarized extremes of macrophages, including M1 macrophages with pro-inflammatory and anti-tumoral activity, and M2-types in contrast, with anti-inflammatory and pro-tumoral functions [2,3,4,18,42,43]. M1 macrophages are activated by lipopolysaccharides (LPS) and the pro-inflammatory cytokines such as interferon-gamma (IFN- γ) [44,45]. They fight against bacterial infections, angiogenesis, and cancer cells, and secrete pro-inflammatory cytokines such as interleukin-1 (IL-1) and tumor necrosis factor (TNF). They have been known as stimulators of adaptive immune responses. In contrast to M1, the M2 macrophages are activated by anti-inflammatory cytokines IL-4, IL-10, and IL-13, and transforming growth factor-beta (TGF-β) [2,4,46]. The diversity of stimuli have generated various subtypes in M2 macrophages [47,48]. M2 macrophages usually inhibit T helper activity and play a key role in promoting tumor progression and neo-angiogenesis events, lacking any cytotoxic activity. Overall, it is the specific signals, and in particular, the cytokines, that determine whether TAMs follow the immune stimulation or immunosuppression functions, and, correspondingly, the promotion or prevention of inflammation and oncogenesis [2,49,50]. It has been confirmed that tumor-derived factors play a singular role in shaping TAM activities in a way that influences cancer progression.

## 3. M1/M2 Switch

Due to the adverse effects of the extreme activity of M1 inflammatory macrophages, a dynamic tendency of the M1 phenotype to switch to the M2-type is often observed in the human body after a while [2,3,4]. Plentiful evidence supports the fact that in the tumor microenvironment, influenced by specific differentiation factors, TAMs tend to get polarized into immunosuppressive M2-types [51,52,53,54,55]. Consequently, M2-types of TAMs promote tumor development and survival [4]. Not surprisingly, M2-types are termed as corrupted policemen of the immune system [4,56]. Although, both the M1- and M2-types are expressed by TAMs simultaneously [57], unfortunately, TAMs routinely act as M2 macrophages [24,58,59,60,61]. Narrowly defined, the term TAM is usually applied to the M2 macrophages located in the tumor tissues which originally come from blood circulation monocytes [62].

## 4. TAM Origin, Recall, and Impacts

Although the precise origin of TAMs is still a debatable question, it has been proposed that macrophages originally stem from bone marrow precursors, circulating monocytes, and one more recently discovered source, splenic reservoir monocytes [6]. Furthermore, some tissue-resident macrophages may rise inside the tumor [63], such as resident macrophages called Kupffer cells in the liver, alveolar macrophages in the lungs, and microglia in the brain [2,49,50]. Studies show that tissue-resident macrophages can be found in nearly all body tissues [64]. However, circulating monocytes provide the greatest source of macrophages in the human body [65].

**Figure 1:**
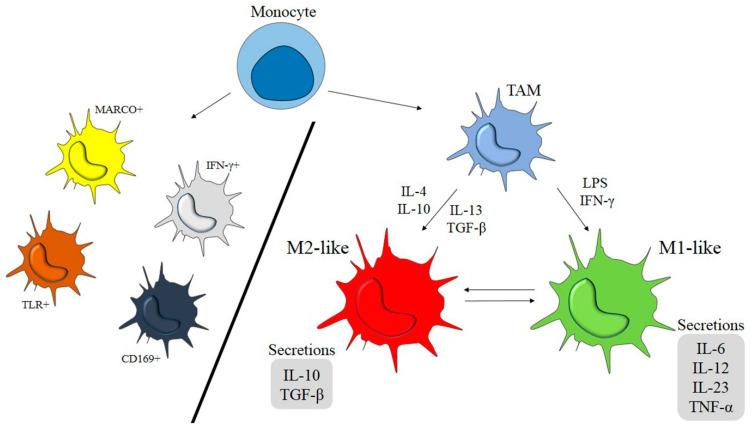
Macrophage polarization model from an original monocyte into different subtypes.

As shown in molecular investigations, the monocyte chemo-attractant protein (CCL2), the macrophage colony-stimulating factor (CSF-1), cytokines, and complement components, are well-known specific signaling molecules that recall inflammatory monocytes to the tumor sites [4,6,63]. Toll-like receptors (TLRs) stimulate macrophages to form M1-polarized types. M1-types are an essential part of the innate host defense system which produces pro-inflammatory cytokines such as IL-1β, IL-6, tumor necrosis factor α (TNF-α), and reactive oxygen/nitrogen species (ROS/RNS) to kill tumor cells [66]. In contrast, IL-4, IL-10, and IL-13 cytokines stimulate M2 macrophage formation. M2-types are necessary for humoral immunity, their anti-inflammatory cytokine secretion, such as IL-10, IL-13, and transforming growth factor-beta (TGF-β), which contribute to cancer development in the human body [67] and also suppress immune system responses [51]. Figure 1 illustrates some important factors involved in M1 and M2 macrophage-related pathways.

The expression of pro-inflammatory and anti-inflammatory metabolites is ultimately reflected in tumor inhibition or promotion. TAM functions can foster or prevent cancer invasion and metastasis. Thus, cancer biology gets profoundly influenced by TAM activity [9,22,31,59,68].

As aforementioned, TAMs play a pivotal role in physiological events in the human body and profoundly affect the cancer inhibition or progression pathways. Since TAM functions are important from a cancer therapy perspective, the following section outlines the significance of TAMs in cancer treatments.

## 5. TAMs Dual Role in Cancer

TAMs encompass both the useful and harmful effects in cancer progression and therapy strategies, which can be explained in the context of many aspects related to their functions [69]. Numerous studies have revealed the positive and negative aspects in this regard, which are outlined in the following sections. Table 1 provides a summary of all of the adversarial and beneficial effects of the different TAM functions in human cancer therapies.

### 5.1. Structural Role

Growing evidence shows that TAMs directly support cancer structural development [74]. In this context, macrophage presence in the tumor microenvironment of human mesothelioma causes rapid and more violent tumor growth [70]. As for human gliomas, around 40 percent of tumor mass belongs to TAMs, which highlights the importance of their presence in the tumor microenvironment [71,72,73]. Mantovani and co-workers have reported that the macrophages may even make up about 50% of solid tumor masses [4].

### 5.2. TAMs Secretions

In addition, investigations illustrate a meaningful correlation between TAMs and the growth and deterioration in different types of cancer. TAMs trigger cancer by signaling molecule production, including growth factors, cytokines, and chemokines [74]. For instance, hepatocyte growth factor (HGF) production by TAMs accelerates hepatocellular carcinoma development [75]. The same pattern of cancer support has been discovered in breast and ovarian cancers and renal cell carcinoma after the macrophage colony-stimulating factor (M-CSF) secretion from TAMs [10]. It has become clear that the TAM-derived growth factors help tumor stem cells (TSCs) to survive. TAM signaling molecules can simulate the self-renewal process, maintenance, and migratory ability of TSCs. Later, TSCs conversely activate TAMs. In fact, TSC-derived factors inspire TAMs to follow pro-tumoral activity. It should be mentioned that TSCs are considered key drivers of tumor initiation and progression that share a common cellular microenvironment with TAMs. However, the precise crosstalk between TAMs and TSCs is poorly understood, but it has been shown that TSC–TAM communication fosters carcinogenesis [76].

### 5.3. Metastasis

TAMs, also play a major part in cancer distant metastasis. Indeed, TAMs facilitate cancer cell migration through the modification in cell–cell junctions and destroying the basal membrane. One of the main destructive activities of M2-type macrophages is their significant contribution to basement membrane and extra-cellular matrix breakdown. This occurs through the presence of an intercellular signaling network derived from TAMs to the top through four major, specific catalytic types of TAM-produced protease enzymes, such as lysosomal cysteine protease [77,78,79], which promote cancer cell invasion in proximity and normal tissues during several sequential steps [78]. For instance, in breast cancer, the cell invasion and lung metastasis happen after cathepsin B production by TAMs [78]. In addition, pancreatic islet cancer metastasis is dependent on cathepsin protease secretion from TAMs [79]. It should be noted that cathepsin plays a significant role in TAM metabolic reprogramming and thus provides remarkable potential in TAM targeting strategies [115].

### 5.4. Resistance Induction

TAMs are not only able to weaken the immune system and foster angiogenesis, cancer invasion, tumor growth, and metastasis, but can also induce resistance to common therapies in some cancers. Impaired macrophage cells cause significant resistance to immunotherapy, chemotherapy, and radiotherapy through TAM-derived metabolite secretion [4,80,81,82,83,84]. The accumulation of evidence correlates the tumor resistance with cytokines released from TAMs. For instance, documents address the impact of TAM-derived IL-6 in drug resistance induction in breast [29], colorectal [85,86], and pancreatic cancers [85]. As indicated by researchers, one of the most important reasons behind immunotherapy failure is the inhibitory checkpoint receptors, such as programmed cell death receptor ligand 1/2 (PD-L1/2), programmed cell death-1 receptor (PD-1), CD80, CD86, and V-domain immunoglobulin suppressor of T cell activation (VISTA), which all derive from macrophages. The inhibition of CD4+ and CD8+ is another consequence of cytokine, chemokine, and enzyme secretion from TAMs which lead to an immunosuppressive status emergence [87,88,89].

### 5.5. Angiogenesis

TAMs are also significant when considered from an angiogenesis perspective. Interestingly, they induce and support the formation of tumor vessels through specific secretion factors such as vascular endothelial growth factor (VEGF), transforming growth factor (TGF-β), interleukin-8 (CXCL8), and platelet-derived growth factor (PDGF). Tumor vessels carrying extra oxygen and nutrients are necessary for tumor growth and progression. [10,69,90]. Even in surgical therapies, the surgery outcome of cancer tissue with a high level of TAMs is worse than tissues with lower TAM numbers [116].

### 5.6. Fundamental Changes

TAMs display various pro-tumoral functions. From a physiological point of view, to keep their high proliferation rate, cancer cells manipulate their cellular metabolism. Taking part in the cancer progression scenario, TAM metabolism reprogramming is in line with cancer progression and metastasis. The TAMs’ specific consumption of oxygen, lipid, glucose, amino acids, and iron nourish their pro-tumoral and immunosuppressive role in cancer development [91,92]. Regarding the TAMs’ more fundamental impacts on cancer development, it is enough to mention the role of TAM-derived factors in cancer stem cell (CSC) preservation and survival. In other words, TAMs can affect tumorigenesis and differentiation from the beginning [93,94,95].

### 5.7. Controversial Role

In some interesting cases, TAMs exhibit a controversial role in cancer progression. An obvious example is lung cancer, in which the CD68+ macrophages’ high attendance in tumor cell islets increases the survival in non-small-cell lung cancer (NSCLC) [12,14,17], while it decreases the survival rate in the lung tumor stroma [12,14,96]. Thus, the prognostic impact of TAMs is both good and bad for tumor islets and tumor stroma, respectively [14]. The overall pattern for lung cancer suggests that high levels of M1 macrophages and resident memory T cells lead to a significantly more satisfying outcome in patients [117].

Furthermore, an extraordinary relationship has been found between acute and chronic pancreatitis with the infiltration of M1-like, pro-inflammatory macrophages and M2-like, anti-inflammatory macrophages, respectively. M2-like macrophage levels have a direct relationship with tumor size and conversely correlates with patient survival [103,118,119].

### 5.8. Prognostic Significance

In breast cancer, there is a relationship between the presence of TAMs and poor prognosis [97,98,99]. Generally, the high-level presence of macrophages has a clear association with a poor prognosis for breast, bladder, prostate, head, and cervical cancers, glioma, melanoma, and non-Hodgkin lymphoma [4,38,100,101,102,120]. Of note, a better prognosis of colorectal and gastric cancer has also been reported as the beneficent side of a high infiltration of macrophages [103,104,105].

### 5.9. Protective Role

As for colorectal cancer, scientists correlate high macrophage infiltration with an increased rate of patient survival [104,106], as well as with a decreased hepatic metastasis rate [107]. This protective role of TAMs in colorectal cancer seems to be the opposite point to their function in other cancers [104]. However, the pro-tumoral characteristic of TAMs has also been proven in colorectal cancer. Based on observations, TAMs can increase the angiogenesis and metastasis rate in some colorectal cancers [114]. Moreover, macrophages also improve the survival rate in patients suffering from osteosarcoma and esophageal tumors [108,109]. Taking TAM advantages into account, they have also expressed great potential in being diagnostic biomarkers in multiple myeloma, esophageal squamous cell carcinoma, and breast, prostate, bladder, lung, pancreatic and gastric cancers [110,111,112,113].

As it is obvious from the literature review and illustrated in Table 1, TAMs may potentially act as a double-edged sword. However, they predominantly seem to be key components in the survival and progression of solid tumors, at least in many cases [121,122,123]. Not surprisingly, TAMs have become a promising therapeutic target for anti-cancer treatment programs [40,84]. Generally, TAM population reduction, macrophages shifting towards the M1-type, and modifying the phagocytosis signaling for more phagocytosis, are considered the main principle of novel therapies designed based on TAMs.

## 6. Therapeutic Targeting of TAMs

Since radiotherapy and chemotherapy are the main current therapies of cancers and cause serious side effects, researchers are optimistic that the modification of the human immune system will provide great opportunities in cancer treatment. TAMs, the singular enriched elements in the TME, are the most important part of novel treatment approaches, the modification of which can strongly overcome cancer disease and defeat drug resistance in mono-therapy frames or in combination with other measures [69].

Current research worldwide addresses several approaches to target TAMs. TAM depletion, macrophage recruitment blocking, reprogramming, phagocytosis signal moderation, and engineering macrophages are the main useful treatment methods that have recently been designed based on TAMs.

### 6.1. TAMs Depletion

To date, several therapeutic approaches have been proposed to remove TAMs from the tumor microenvironment. Studies have reported the increase in efficiency of some drugs after macrophage depletion [40]. In other words, the inhibition of TAM accumulation in the tumor microenvironment is a useful approach in the fight against cancer progression [41]. Significantly, the tumor-associated macrophage depletion has unexpectedly been observed in patients with a satisfactory treatment outcome after receiving trabectedin, a recently approved anti-cancer agent. Now, the removal of macrophages is demonstrated as an important component of the trabectedin mode of action and a promising approach to halt cancer progress [124]. The anti-tumor effect of TAM depletion has also been reported for primary and metastatic melanoma treatment [125]. In addition, TAM depletion helps in cervical and breast carcinoma treatment as well [16]. TAM population reduction is an effective measure for primary and metastatic melanoma treatment [125]. Clodronate-loaded liposomes are another compound that reduces the TAM population [125]. The chemicals mentioned reduce the TAM population by inducing apoptosis in mononuclear phagocytes [126,127]. Some specific antibodies are also appropriate options to remove TAMs and cure colorectal cancer [128] and glioma tumors [129]. TAM depletion significantly increases the anti-cancer effects of sorafenib and inhibits tumor progression and angiogenesis, as well as the lung metastasis of liver cancer [130]. Meanwhile, TAM blocking by the CSF-1 receptor inhibitors or CSF-1 antibodies can break prostate cancer resistance [131] and reverse tumor progression in mammary tumors [132].

### 6.2. Recruitment Blocking

There is also a potential to inhibit the monocytes’ differentiation into macrophages, or in fact, to prevent macrophage recruitment, which is possible through the modification of related chemokines [133,134,135]. Some measures have been examined in this regard, including the CCL2 blocking that reduces monocyte recruitment and macrophage infiltration which leads to tumor growth inhibition [136,137,138]. Carlumab usage, which is an anti-CCL2 monoclonal antibody, has successfully decreased the tumor growth in prostate cancer [136]. In animal models, CCL2 blocking has been effective on glioma, colon cancer, prostate cancer, and melanoma [134,139]. Inhibition of CCL2 decreases TAM population and can also be used in combination with chemotherapies. However, complementary clinical studies are under investigation [140]. In addition, the application of macrophage colony-stimulating factor (M-CSF) inhibitors can effectively reduce TAMs and prevent tumor growth and proliferation in pancreatic cancer in mouse models [141,142,143]. The M-CSF inhibition exhibits similar positive results in terms of TAM reduction in glioma mouse models [141,144,145]. It should be mentioned that the M-CSF and its related receptors boost TAM interaction with tumor cells [146].

### 6.3. TAMs Reprogramming

TAMs are powerful weapons in the fight against cancer. Some new treatment methods have relied on using TAMs themselves as therapeutic equipment. The term “reprogramming” often comes across in these approaches, which is defined as an effort to switch pro-tumoral and immunosuppressive M2-type macrophages towards anti-tumoral M1-types [39]. The reprogramming aim is to revert the immunosuppression of TAMs and to restore/release their anti-tumoral functions [39]. The M2 macrophages trans-differentiating to M1 results in adaptive immune response triggering [147,148,149,150]. It has been demonstrated that reprogramming boosts the effectiveness of current anti-cancer therapies like implementing immune-checkpoint inhibitors [83]. During reprogramming, the M2-type manipulation towards M1 is usually performed through the modification of specific signals, which regulate the M1/M2 shifting pathway [4]. However, now, various approaches have been introduced to implement the reprogramming of tumor-associated macrophages, namely the signaling activation of toll-like receptors (TLR) and the stimulator of interferon genes (STING), using monoclonal antibodies, genetic and epigenetic intervention (i.e., the interference RNA application to silence the expression of immunosuppressive genes and activate the stimulatory pathways in macrophages), and also metabolic manipulation (i.e., the interference macrophage metabolic pathways) [39].

To induce reprogramming, some studies have suggested inducing pro-inflammatory cytokines to boost immune system responses to cancer cells [149,150,151]. The over-secretion of cytokines, such as IL-12, IL-23, and IL-8, can control many tumor growths [152]. The positive effects of such approaches have been demonstrated in patients suffering from metastatic melanoma and also non–small-cell lung cancer [153]. In addition to this, the modification in specific receptors, such as TLRs and the stimulator of IFN gene (STING), can ultimately result in TAM reprogramming to the pro-inflammatory phenotype [154,155,156,157]. The knockdown of pro-inflammatory signaling pathways with the RNA silencing method has been reported to be an effective measure for macrophages shifting from M2- to M1-types [158,159,160]. As another approach, macrophage metabolism moderation occurs by M1 macrophage activation along with M2 macrophage suppression [147,161,162].

Reprogramming has shown a significant reduction in tumor size in pancreatic cancer mouse models [1,163]. Additionally, TAM reprogramming to dendritic cells can remove the tumor cells in glioma mouse models [164]. Exploring the combination of TAM reprogramming with other pharmacological strategies, such as chemotherapies, anti-checkpoint inhibitors, or adoptive cell transfer, may provide an even more promising future in cancer therapies [39,40].

**Figure 2:**
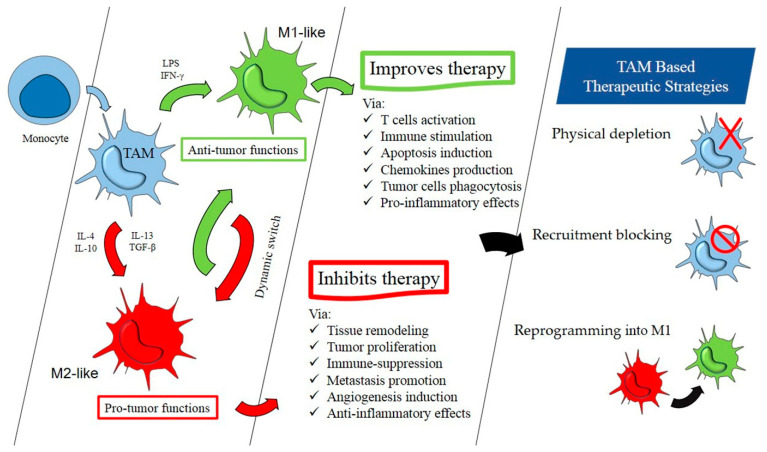
M1- and M2-like macrophage significance in cancer treatment.

As can be seen from the literature review, a wide range of therapeutic measures inspired by TAM targeting are under research and development. Significantly, the TAM targeting measure may lead to even better outcomes when combined with other therapies like radiotherapies and chemotherapies [28,136,140,165]. Considering the therapy resistance or side effects of radiotherapies and chemotherapies, the TAM-mediated treatment strategies will open a novel avenue of well-adapted therapies. After years of relentless research, the associated community hopes this way more effective treatments for cancer elimination will appear. Figure 2 schematically summarizes the information regarding the origin, dual role, and conflicting therapeutic impacts of two extremes of TAM polarization, in addition to their potential in cancer treatment strategies.

## 7. Treatment Drawbacks

TAMs, not only by their presence and functions, foster serious drawbacks in the efficacy of anti-cancer treatments but their targeting strategies are also faced with specific limitations as well. There are three major concerns regarding the negative effects of TAMs on cancer therapy.

The first problem addresses TAM interference in common cancer therapies. It has become clear that TAMs can potentially determine how cancer cells respond to chemotherapy, radiotherapy, and immunotherapy treatments. TAMs are considered great synergistic or antagonistic counterparts for common therapy methods. They recruit a wide range of mechanisms to cripple chemotherapy treatment effectiveness. For instance, in response to the anti-cancer agent’s application, macrophages, in a compensatory action, trigger the process of a tissue repair response which promotes cancer cell survival. This misdirected response is being interpreted as a strong obstacle to the anti-cancer effects of pharmaceutical agents [144,166,167]. The tumor-protective function of TAMs has been shown in mammary tumor protection against PTX, doxorubicin, and etoposide drugs [20,168], cervical cancer survival despite platinum application [166], and pancreatic cancer escape from gemcitabine agent [169], to name a few. Indeed, macrophages protect cancer cells against anti-cancer drugs by cytokine secretion, as well as other ways. Today, it has been demonstrated that TAMs are able to regulate cancer cell responses against anti-cancer drugs [141,170]. The physical presence of TAMs in tumor sites, their polarization into pro-tumoral forms, and specific metabolite secretion from them, are considered as the most remarkable mechanisms behind their negative effects on chemotherapy measures [20,166]. However, in contrast, some investigations reveal the positive effects and supportive role of TAMs in chemotherapy trials [40]. Similar to chemotherapy, the profound influence on radiotherapy due to TAM functions has yet to be found. Reportedly, TAM infiltration can strongly reduce radiotherapy efficiency through various mechanisms [28,171,172,173,174]. Likewise, some positive effects of TAM functions on radiotherapy outcomes have also yet to be disclosed [40]. Surprisingly, TAMs are also able to interfere with immunotherapy results through different mechanisms. For example, they can hamper the high efficacy of immune-checkpoint blockade therapy [88,89,175]. Studies have shown that TAMs can also ruin anti-angiogenic therapy effects. They can foster resistance to anti-angiogenic therapy in some tumors such as murine glioblastoma [176,177]. As can be seen from previous investigations, TAMs have a dramatic impact on the outcomes of common therapies, and vice versa, which, in consequence, treatment improvement happens just occasionally, but therapy resistance or poor treatment outcomes are prevailing. Scientists hope that understanding the detailed interactions between TAMs and common cancer therapies will pave a better way to reduce cancer in patients.

Secondly, TAM targeting strategies are not devoid of their limitations. Although TAMs have a profound influence on cancer progression, and thus have a become direct target in some cancer therapy measures [69], the execution of two general strategies, including the inhibition of TAM infiltration into the cancer environment (through recruitment prevention or physical depletion), and TAM reprogramming using pharmaceutical agents and approaches, are not completely flawless [167]. Although limited results may disappoint the agent’s application, the high toxicity for non-cancer cells, and not enough specificity of drugs used for targeting TAMs, are being mentioned as serious side effects of TAM-based therapies [165]. Meanwhile, TAM targeting seems to be unable to eradicate cancer by itself and is toxic for patients at inordinate applications/dosages [178]. Undoubtedly, more future attempts are needed to define the various TAM subpopulations in different human cancers and to execute cancer- and patient-specific TAM-based therapies to gain more conducive treatment results. Overall, it is too important to bypass unsatisfactory and unintended clinical results in the future.

Last but not the least, the unknown outcomes of TAM modification, encompass the third concern. Indeed, another important problem with TAM targeting is the obscure effects of their modification of human physiology and metabolism. Unquestionably, the complexity of mechanisms behind TAM impacts on cancer progression and metastasis, immune system response, and patient survival, is beyond our expectations. Thus, any alteration in TAMs may potentially result in unknown complications. This is why the role of TAMs in cancer therapy still needs to be explored in-depth. Specialists expect further investigations to shed light on the dark side of the complexity of TA-related mechanisms in cancer progression and metastasis.

As a conclusion, the literature review conveys the opinion that (1) however impressive, the macrophage-targeting strategy in itself seems to be insufficient for cancer treatment, and (2) TAMs significantly contribute to the failure of therapeutic approaches through drug resistance, hence, taking the role of TAMs in the patient’s destiny into account, it seems to be an indispensable part of therapies to regulate TAM functions in the future. However, TAM-based cancer therapies are obviously in their infancy, and we are prompted more than ever before to explore how their modification might impact cancer therapy methods, and how it can improve the effectiveness of common treatments. To date, the most valuable lesson is not to extend the preclinical experiment results of TAM-targeting to clinical trials without enough insurance of safety and effectiveness, otherwise, the treatment approaches will immediately be prone to failure.

## 8. Novel Therapy Approaches

Currently, a broad clinical trial of TAM-based cancer therapy has not been utilized anywhere in the world, however, data obtained from experiments on animal models and preclinical investigations hold the promise that they can be used as anti-cancer targets in the future. In addition to the discussed anti-cancer potential of TAMs, there is also another benefit provided by TAMs in the fight with human cancer; an approach has been developed to deliver anti-cancer drugs to cancer cells.

During the last decade, some novel cancer therapy methods have received significant attention. Among them, numerous studies suggest a strong link between nanoparticles and TAMs in cancer therapy measures. In this context, nanoparticle application has been developed to improve drug transportation to the target cells. Previously, the usage of ultra-small polymer nanoparticles has been reported to be effective in thymoma and murine melanoma treatment through myeloid-derived suppressor cells (MDSC) and a macrophage depletion program [179]. Previously, the careering potential of TAMs in nanoparticle delivery into gliomas had been demonstrated by Alizadeh and colleagues. Their study mentioned the possibility of drug uptake by TAMs and their potential in cancer therapy [180]. Before that, Choi and co-workers had proposed the potential of macrophages in nanoparticle-based drug delivery into tumor regions. They explored several therapeutic strategies in this regard and monitored the distribution of gold-conjugated macrophages within the tumor region as well [181]. It has been well-established that TAM phagocytosis potential and movement have made them successful candidates in nanoparticle-based drug delivery into cancer cells. Thus, TAMs are taking a significant place in novel cancer treatment strategies. In the frame of monotherapy or in combination with other treatment methods, we expect to witness the advance in novel drug delivery approaches which improve TAM-based treatments.

Additionally, some investigations address the potential of nanocarrier application to reduce the expression level of TAMs themselves as a cancer treatment method. During an exploration performed by Zhang and co-workers (2015), they developed a glucan-based siRNA carrier system in non-viral nanoparticles to reduce the migration inhibitory factor (MIF) gene expression level in TAMs [182]. The idea of delivering siRNA nanoparticles into macrophages, and its promising results in both in vitro and in vivo trials, keeps our hopes alive to use macrophage-targeted siRNA to fight cancer. Overall, due to their physicochemical characteristics, nanocarrier application may ensure the high efficacy, specificity, and safety of drugs [183,184]. However, TAM interaction with nanoparticles, and its significance in cancer therapy, remains to be explored in detail.

As another novel approach, macrophage modification at transcriptional, epigenetic, and metabolic levels has received significant attention recently and taken a significant place in anti-cancer strategy development. In this regard, the significance of transcription factors, such as PU.1, STATs, NF-kB, c-Myc, IRFs, SNAILs, and Mafs, in macrophage polarization in the tumor microenvironment has yet to be widely explored. In addition, DNA methylation, histone modifications, and microRNA application are named as regulatory approaches for TAM alteration at the epigenetic level. It has been thought that the additional studies in TAM alteration at the transcriptional, epigenetic, and metabolic levels may show a promising new horizon in the fight with many cancer types such as breast, colon, colorectal, lung, ovarian, pancreatic, prostate and skin cancers, glioma, myeloma, hepatocellular carcinoma, and many others as well [185,186]. Table 2 summarizes the most promising TAM-based novel therapy approaches and their involvement in specific cancers and their therapies.

In line with novel therapy approaches, many ongoing investigations are expanding our understanding and overall knowledge around the potential of TAMs in cancer therapy. For instance, some recent studies address the interference of TAM subpopulation activity using specific antibodies injection. It has been shown that scavenger receptor MARCO antibodies can stop TAM-mediated immunosuppression and serves cancer immunotherapy [198]. According to the literature review, most of the novel TAM-based therapies usually rely on the natural characteristics of tumor or cancer cells, the immune system, and the tumor microenvironment [199]. However, the different TAM subpopulations, and the various recruitment and evolution process, which is raised from their regional differences, justifies the necessity of multi-targeted approaches for the implementation to fight against cancer [200]. The investigations performed, mostly in the last five years, leave room for bright and propitious treatment results in the future [201].

## 9. Conclusions and Future Perspectives

Since tumors are still a great life threat for many, expanding the knowledge on the reactions of the immune system in the disease is crucial to building new and precise methods of treatment. The influence of TAMs on the development of cancer may be realized in multiple ways. Since their role in cancer progression is not yet discussable, they are currently thought to be of potential use for future immunotherapy research methods, including the use of macrophage secretome. Macrophages may serve as a promising tumor-targeted therapy, but further investigations are needed to fully understand the potential of these cells in anti-tumor responses. Finally, as described previously, several novel and promising cancer therapy approaches have yet to be achieved that may potentially revolutionize cancer therapy methods. Taking the limitations of common therapies into account, now that the molecular approaches are the pioneer of novel treatments, it is time to lead a wide range of projects that cover both the molecular approaches and advanced measures of drug delivery. This way, more satisfactory outcomes from patient treatment programs might be witnessed in the future.

## Figures and Tables

**Table 1:** Adversarial and beneficial effects of the TAM functions in several cancer therapies.

TAM Function	Mechanism	Outcome	Cancer Type	Therapeutic Impact	References
Structural role	Physical presence	Rapid and violent tumor growth	Gliomas, solid tumors	Negative	[70,71,72,73]
Secretions	Signaling molecules, growth factors, cytokines, chemokines	Cancer initiation, tumor growth, cancer cells survive	Hepatocellular carcinoma, breast and ovarian cancers, renal cell carcinoma	Negative	[10,74,75,76]
Metastasis promotion	Protease enzyme modification in cell–cell junctions and basal membrane	Distant migration and invasion of cancer	Breast cancer, pancreatic islet cancer	Negative	[77,78,79]
Resistance induction	Metabolite secretion like cytokines, PD-L1/2, PD-1, CD80, CD86, VISTA	Resistance to common therapies (immunotherapy, chemotherapy, radiotherapy)	Breast, colorectal and pancreatic cancers	Negative	[4,29,80,81,82,83,84,85,86,87,88,89]
Angiogenesis	VEGF, TGF-β, CXCL8, and PDGF secretion	Formation of tumor vessels, tumor growth, and progression	Mammary tumors, osteosarcoma	Negative	[10,69,90]
Metabolism reprograming	Pro-tumoral and immunosuppressive effects of TAM-derived factors	Tumorigenesis and differentiation, cancer progression, metastasis	Ovarian carcinoma, prostate cancer, cervical cancer, breast cancer	Negative	[91,92,93,94,95]
Dual role	High attendance and infiltration	Patient survival decreases	Lung tumor stroma	Negative	[96]
		Patient survival increases	Non-small-cell lung cancer	Positive	[12,14,17]
		Poor prognosis	Breast, bladder, prostate, head, and cervical cancers, glioma, melanoma, and non-Hodgkin lymphoma	Negative	[4,38,97,98,99,100,101,102]
		Good prognosis	Colorectal and gastric cancer	Positive	[103,104,105]
Protective role	High infiltration	Patients survival increase, decreased metastasis	Colorectal cáncer * osteosarcoma, esophageal tumors	Positive	[104,106,107,108,109]
Diagnostic role	Diagnostic characteristics	Diagnosis and therapy improvements	Multiple myeloma, esophageal squamous cell carcinoma, breast, prostate, bladder, lung, pancreatic and gastric cancers	Positive	[110,111,112,113]

* In some colorectal cancers, the pro-tumoral effects, angiogenesis, and metastasis increase have also been proved as negative effects of TAM functions [114].

**Table 2:** Novel cancer therapy approaches developed based on TAM functions.

Approach	Mechanism	Cancer Type	References
Nanoparticles	Drug transportation for macrophage depletion	Thymoma and murine melanoma	[179]
	Drug delivery into tumor regions by TAMs	Glioma and human breast tumor	[180,181]
	Reduction in TAM expression level	Mice mammary tumors	[182]
TAMs molecular modification	Modification in transcription factors	Metastatic melanoma, breast, ovarian, lung, and colorectal cancers and glioblastoma	[54,187,188,189]
	DNA methylation	Colon cancer, melanoma, and murine model of non-small lung cancer	[190,191]
	Histone modification	Breast and prostate cancers, mouse models of pancreatic and lung cancers	[187,192,193,194]
	MicroRNAs application	Human gastric cancer, hepatocellular carcinoma, cervical carcinoma, breast and lung cancer	[195,196,197]

## Data Availability

Not applicable.
